# Transcriptome Profiling of the Murine Testis during the First Wave of Spermatogenesis

**DOI:** 10.1371/journal.pone.0061558

**Published:** 2013-04-17

**Authors:** Asta Laiho, Noora Kotaja, Attila Gyenesei, Anu Sironen

**Affiliations:** 1 The Finnish Microarray and Sequencing Centre, Turku Centre for Biotechnology, University of Turku and Åbo Akademi University, Tykistökatu 6, Turku, Finland; 2 Agrifood Research Finland, Biotechnology and Food Research, Animal Genomics, Jokioinen, Finland; 3 Department of Physiology, Institute of Biomedicine, University of Turku, Turku, Finland; University Hospital of Münster, Germany

## Abstract

Correct gene expression patterns form the basis for male germ cell differentiation and male fertility. Although previous studies have elucidated the importance of testis specific gene expression, the exact transcripts and comprehensive gene expression patterns remain unknown. Large scale sequencing techniques have enabled cost effective analysis of gene expression and isoform studies. Using the SOLiD 4 next-generation sequencing platform we have investigated the gene expression patterns at five different time points during the first wave on murine spermatogenesis. Our results highlight the upregulation of spermatogenesis related biological processes and associated cellular components. Elucidation of differential gene expression at important time points during the sperm development emphasizes the importance of correct timing of gene expression within biological processes. Differential gene level expression was analyzed with R/Bioconductor’s Limma package and isoform analysis was conducted with the Cufflinks pipeline. At gene level total of 2494 differentially expressed genes were identified and Cufflinks characterized over 160 000 gene isoforms, of which 29% were novel transcripts assigned to known genes. Isoforms were detected for 57% of expressed genes and in a total over 26 000 genes were expressed in the testis. Differential promoter and transcription start site usage appears also to play a role in regulation of gene expression during spermatogenesis. Furthermore, we identified 947 upregulated long non-coding RNAs during the first wave of spermatogenesis. These RNAs appeared to be highly specific to different time points. Transcriptomic analysis of testis tissue samples is highly informative due to the large number of expressed genes and identified isoforms. Our study provides a very valuable basis for investigation of gene isoforms and regulation and factors contributing to male fertility.

## Introduction

Spermatogenesis is a complex process, where spermatogonia develop into highly differentiated spermatozoa through several strictly controlled steps. Mouse spermatogenesis begins by mitotic proliferation of type A, Intermediate and type B spermatogonia. Type B spermatogonia then divide to form early spermatocytes, which marks the beginning of the meiotic phase of spermatogenesis. After the long-lasting prophase of the first meiotic division, the remainder of the cell division process is completed rapidly and two subsequent divisions produce haploid round spermatids. In the last differentiation phase (spermiogenesis), spermatids undergo dramatic cell transformation that includes chromatin condensation and nuclear shaping, removal of excess cytoplasm, and the acrosome and sperm tail formation. Finally, mature spermatozoa are released into the lumen of seminiferous epithelium and transported to the epididymis for further maturation. Development of spermatogonia to haploid spermatids takes approximately 35 days in mice [1], of which the spermiogenic phase lasts about 2–3 weeks [2].

The first wave of spermatogenesis in mouse is initiated only a few days after birth and proceeds in a synchronized manner. Key time points for appearance of particular germ cell types are well defined during this first cycle of spermatogenesis. Seven-day-old mouse testes contain only Sertoli cells (SCs) and spermatogonia in the seminiferous tubules. At postnatal day (PND) 9 early spermatocytes appear, at PND 14 the pachytene stage of the first meiotic prophase is initiated, and at PND 18 late pachytene and diplotene spermatocytes are also present. As a result of meiotic divisions, round spermatids appear around PND 20 and at PND 30 spermatids have already reached the elongation phase and sperm tail accessory structures are being constructed [3]. Due to the synchronized progression of the first wave of spermatogenesis, it provides an excellent model system to study gene expression during sperm development.

Gene expression during spermatogenesis is highly orchestrated and strictly regulated at transcriptional and posttranscriptional level. In order to complete the very complex development of spermatozoa, several specific transcriptional regulators are needed. Transcription is regulated both by epigenetic chromatin modifications and trans-acting factors that bind to the specific DNA sequences in the promoter regions [4]–[6]. In late elongating spermatids transcription is largely silenced as a consequence of the chromatin condensation by histone-protamine transition. Most of the RNAs found in male germ cells during this phase are produced earlier and are translationally regulated in specific ribonucleoprotein particles [2].

Previous studies of large scale gene expression in spermatogenesis have been conducted with microarray experiments. Several studies have been dedicated to the identification of gene expression differences in normal versus fertility defected patients (reviewed in [7]). In addition, serial analysis of gene expression (SAGE) and microarray profiling of rodent testis samples and enriched cell populations have identified a large number of genes important for spermatogenesis [8]–[15]. The most comprehensive map of the mammalian testicular expression program at a cell-type specific level identified 7066 genes, which were classified into somatic (1,684), mitotic (2,581), meiotic (1,809), and postmeiotic (1,435) expression clusters, however this classification does not necessarily reflect cell-type specific expression [16].

Although these and single gene expression studies have been able to elucidate the stage specificity and complexity of testicular transcription machinery [10], the overall picture of the gene expression patterns and transcript variety during spermatogenesis is still largely unknown. Over 50% of all known mouse genes have been shown to be expressed during testicular development and over 47% of the expressed transcripts are uncharacterized [17]. In addition, it has been evaluated that ∼30% of the murine genome is differentially expressed during testis development. Major periods of expressional change occur during the first few days after birth (PND 0–6), at the beginning of meiosis (PND 12–14), and along with the appearance of haploid gametes (around PND 20) [14]. Microarray experiments are limited to known transcripts and the analysis is hindered by cross-hybridization and high signal to noise ratio. Next-generation sequencing (NGS) has enabled the extensive investigation of gene expression without any prior knowledge of transcript content in the sample. NGS can also identify and quantify rare transcripts and provides information regarding alternative splicing and sequence variation in identified genes.

In this study, we have analyzed the gene expression differences during the first wave of murine spermatogenesis. Total testis tissue samples at critical time points during germ cell differentiation were collected at PND 7, 14, 17, 21 and 28. Since proper development of male germ cells requires correct function of testicular somatic cells and differences in gene expression may be affected by cell-cell interactions, the whole testis is a desirable sample type for investigation of gene expression during spermatogenesis. Replicate samples were sequenced with the SOLiD 4 platform and analyzed for detection of differential gene expression and splicing variants. In addition, functional annotation of identified gene expression differences highlighted biological processes important for correct sperm development.

## Materials and Methods

### Ethics Statement

All mice were handled in accordance with the institutional animal care policies of the University of Turku. Mice were maintained in a specific pathogen-free stage at the Central Animal Laboratory of the University of Turku and sacrificed by CO_2_ inhalation. The studies were approved by the Laboratory Animal Care and Use Committee of the University of Turku.

### Animals and Sample Preparation

Whole testes from two C57BL/6NHsd mice (Mus musculus) of each time point were collected and snap frozen in liquid nitrogen and stored at −80°C prior to RNA extraction. The presence of specific cell types in the testis samples was confirmed with squash preparations of the pieces of the seminiferous tubules followed by phase contrast microscopy. A fraction of the testes was dissected and short tubule segments were cut in PBS [18]. Tubule segments were transferred with a pipette on microscope slides in 15 µl of PBS. A coverslip was placed carefully onto the tubule segment, and the excess fluid was removed by blotting, which allowed the cells to float out from the tubule.

### RNA Extraction and Library Preparation

Total RNA was extracted using RNeasy Midi kit (Qiagen) following the manufacturer’s instructions. The quality and concentrations of the RNA was checked with Agilent's 2100 Bioanalyzer (Agilent) and Nanodrop ND-2000 spectrophotometer (Thermo Scientific). Ribosomal RNA was removed with RibominusTM Eukaryote Kit for RNA-Seq (Invitrogen) and ribosomal RNA-depleted total RNA was fragmented using RNaseIII, to convert the whole transcriptome sample to RNA of a size appropriate for SOLiD™ System sequencing. After cleanup using the PurelinkTM RNA Micro kit, fragmented RNA samples with sufficient yield and an appropriate size distribution were ready for preparation of amplified cDNA libraries. Quality of the fragmentation was checked with Bioanalyzer. The fragmented RNA sample was hybridized and ligated with the Adaptor Mix. RNA population with ligated adaptors was reverse transcribed to generate single-stranded cDNA copies of the fragmented RNA molecules. After a cleanup step using the MinElute® PCR Purification Kit, the sample was subjected to denaturing gel electrophoresis, and gel slices containing cDNA in the desired size range were excised. The size-selected cDNA was amplified using 15 cycles of PCR that takes place in the gel slices. This step appends required terminal sequences to each molecule and generates sufficient template for SOLiD sequencing. After the PCR, the amplified cDNA was cleaned up using PureLinkTM PCR purification kit. Libraries were quantitated with two different methods; Qubit fluorometer (Invitrogen) and quantitative PCR to warrant the accuracy. SOLiD Library TaqMan Quantitation Kit was used for determining the molar concentration of amplified template in a SOLiD library. In qPCR, the standard and unknown library template are amplified using two sequence-specific primers with a TaqMan fluorogenic probe labeled with FAM™ dye and a dye quencher. The uniformity of fragment size of libraries was confirmed with Bioanalyzer. Templated bead preparation was performed by emulsion PCR (ePCR). SOLiD™ EZ Bead™ instrumentation was used for templated bead preparation.

### Transcriptomic Data Analysis

The colorspace reads obtained from the SOLiD sequencer were aligned against the mouse reference genome (mm9 assembly) using the standard whole transcriptome pipeline and the colorspace alignment tool provided by Applied Biosystems and distributed with the instrument (LifeScope v2.1). Reads associated with ribosomal RNA, transfer RNA, repeats and other uninformative reads were filtered out during the process as well as reads with more than 10 potential alignments.

After alignment to the reference genome, the unique reads were associated with known genes based on Ensembl annotations, and the number of reads aligned within each gene was counted. The counts are reported as RPKM (Reads Per Kilobase of exon model per Million mapped reads) values, which are used for normalizing the count values by taking the gene lengths into account. The values produced are also independent of the total number of the reads in each sample and thus make the data comparable across the sample set.

Quality control for the data was performed using R/Bioconductor and R-package Limma was used for performing the statistical testing between the groups. Limma was tested with various data normalization and transformation methods, but the choice of preprocessing methods did not have a significant effect in the ranking of the result genes. The sample groups were compared in chronological order. P-value of at least 0.01 and an absolute fold-change of at least two was required for a gene to be called significantly differentially expressed between the groups. It was also required that the samples had at least 10 unit difference between the group mean expression values. TopGO and GOstats R/Bioconductor packages were used for functional enrichment analysis of the differentially expressed filtered gene lists towards KEGG pathways and Gene Ontology categories. Heat maps comparing the enrichment results across comparisons were produced with GENE-E tool (http://www.broadinstitute.org/cancer/software/GENE-E/) using pearson’s correlation metrics and average linkage distance metrics.

Gene variant detection and differential transcript expression analysis were carried out using the Cufflinks pipeline [19]. The LifeScope mapping results were used as an input also for the Cufflinks analysis. In the Cufflinks analysis the counts are reported as FPKM (Fragments Per Kilobase of exon model per Million mapped reads) values. GO enrichment analyses for Cufflinks results were carried out using GOrilla [20], [21].

## Results

### Data Evaluation

The samples were sequenced with the SOLiD 4 instrument at 50 bp read length. In total 10 samples were used for the study. Samples were collected at different time points during the first wave of mouse spermatogenesis. Each time point corresponds to specific cell content in the testis. First time point was at PND 7, when only somatic cells and spermatogonia are present in the testis. Thereafter additional cell types are present in selected samples in chronological order: PND 14 contains early spermatocytes, PND 17 late spermatocytes, PND 21 round spermatids and finally PND 28 elongating spermatids. The assumed cell content in each sample was also confirmed by phase contrast microscopy of squash preparations (Figure S1A). The analysis of squash preparations confirmed the expected and similar cell content in each sample group. In addition, the testicular weights within the groups were highly comparable (Figure S1B). Each time point was analyzed in two biological replicates and PND 7 samples were divided to two barcodes for technical reasons (Table 1).

**Table 1:** SOLiD 4 read counts and mapping statistics for individual samples.

Sample	Total reads	Mapped reads	Mapping rate	Total reads passing filters	Counted onexons	Counted on introns	Counted on intergenic regions
PND_7_s1	45,706,071	21,230,399	47%	14,518,593	9,476,631	4,001,849	1,040,113
PND_7_s2	46,592,933	16,249,678	35%	10,811,100	6,911,439	3,074,225	825,436
PND_14_s1	33,787,664	16,249,484	48%	11,373,935	7,657,560	2,599,696	1,116,679
PND_14_s2	26,776,971	13,525,537	50%	8,652,432	6,946,490	1,037,577	668,365
PND_17_s1	54,374,927	25,471,965	46%	15,825,273	13,133,830	1,490,487	1,200,956
PND_17_s2	16,733,599	7,235,322	43%	4,611,709	3,890,612	404,87	316,227
PND_21_s1	26,409,512	8,475,148	32%	5,700,864	3,961,522	1,006,336	733,006
PND_21_s2	23,544,136	11,445,588	49%	7,403,489	6,150,493	635,138	617,858
PND_28_s1	29,895,585	15,482,573	52%	10,513,142	8,709,227	814,463	989,452
PND_28_s2	23,640,573	9,106,849	39%	6,141,624	5,064,489	467,358	609,777

Total number of reads varied from 18 to 54 million, the mapping rate of filtered reads being 32–52% (Table 1). Most of the reads were mapped on exons (50%), although a high number of reads was also mapped on introns or intergenic regions (Table 1). Some differences in read counts were observed between biological replicates, especially between PND_17_s1 and s2. The starting material (amount of total RNA) was identical in these samples, thus the differences arise possibly from the sample preparation and Solid run. However, the difference in total read counts do not significantly affect the expression analysis, since the RPKM and FPKM values are corrected with the total read count for each sample. Visualization with the IGV confirmed the even distribution of reads throughout the genome and at exonic regions (data not shown).

For gene expression analysis, only those features were used, which had non-zero count values among the sample set (n = 26 789 genes). The results for total and mapped reads, mapping rate, total reads passing filters, counted exons, introns and intergenic regions are presented in Table 1.

### Sample Correlations

**Figure 1:**
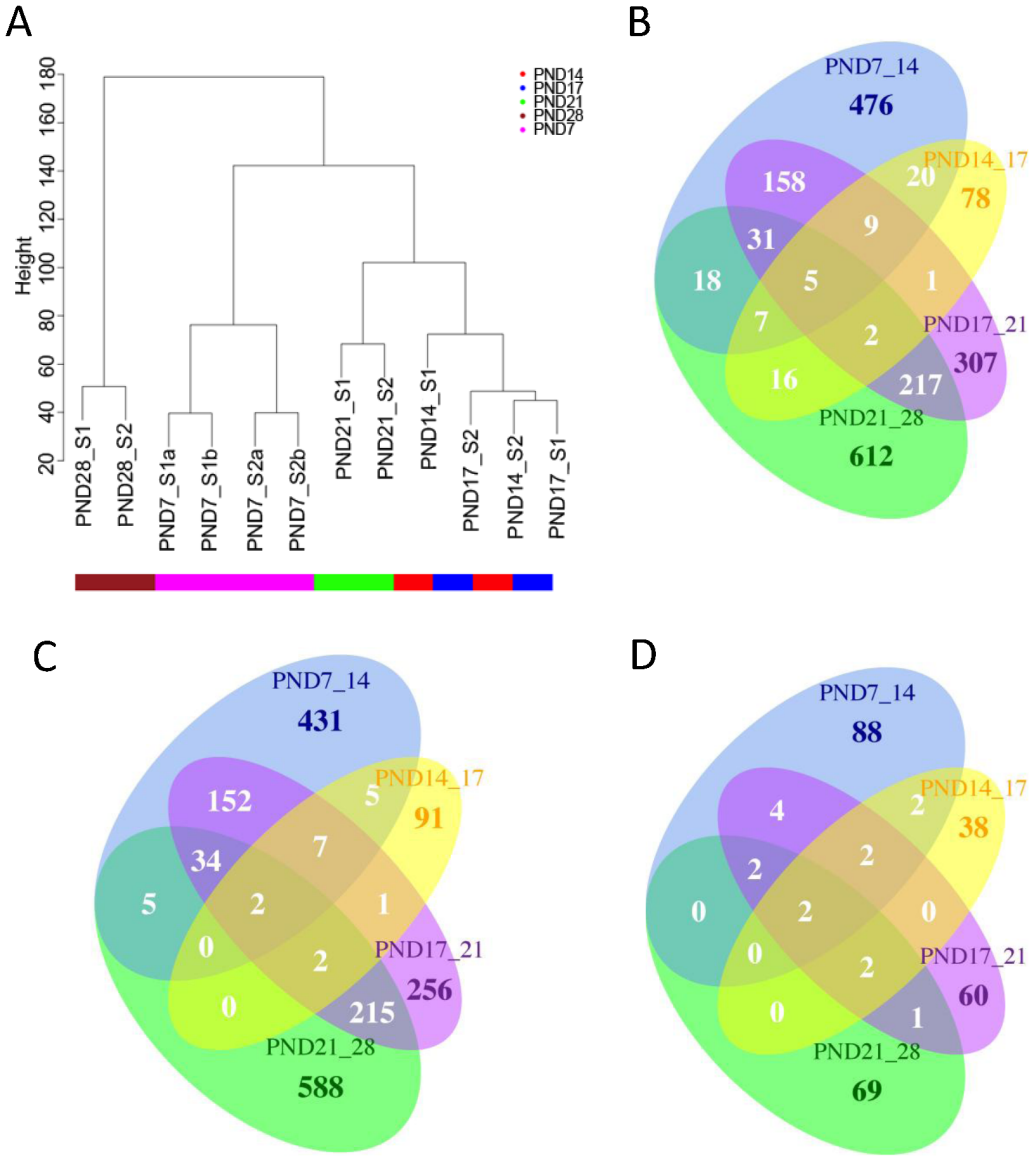
Clustering of sample groups and overlapping DE genes. A. Hierarchical clustering for the normalized data. Biological replicate samples cluster closely together. Sample groups PND28 and PND7 are more distant to other groups and PND14 and PND17 are similar to each other based on the gene expression differences. B. Total number of overlapping DE genes between comparisons. C. Agreeing and overlapping upregulated genes between comparisons. D. Agreeing and overlapping downregulated genes between comparisons.

Pearson’s metrics was used to measure the similarity between samples. Correlations between replicate samples varied between 0.97 and 0.99 depicting very high reproducibility. The samples clustered according to the sample groups. PND 14 and 17 sample groups were highly similar to each other. Sample groups PND28 and PND7 were more distant to other groups. Figure 1A shows the hierarchical clustering of the individual samples.

### Comparisons of the Sample Groups

The sample groups were compared in chronological order using the R-package Limma. Comparison of PND 14 and PND 17 samples had the lowest amount of differentially expressed (DE) genes (Table 2) consistent with the results obtained with hierarchical clustering (Figure 1A). A high number of DE genes were present between PND 7/14 and 17/21 and 21/28 (Table 2) and most of the identified DE genes were specifically regulated in one comparison (Figure 1B). Highest difference in gene expression was detected between samples PND21 and PND28 during spermatid differentiation. Total number of 906 DE genes were found between these time points, and 612 of them were differentially expressed only in this comparison (Table 1 and Figure 1B). These results correlate with the expected pattern of gene expression during spermatogenesis. The most prominent morphological and biochemical changes in sperm development occur during spermatid differentiation starting from PND 20. Transcription of genes required for the construction of sperm-specific structures starts already in late spermatocytes and continues throughout the round spermatid phase and in early elongating spermatids. Most of the transcripts needed during the late steps of spermiogenesis have to be present in early elongating spermatids at PND 28 before the cease of transcription in condensing nucleus.

**Table 2:** Total number of Ensemble annotated DE genes and up- and downregulated gene numbers between sample groups.

	Tot	Up	Down
PND7/PND14	724	632	92
PND14/PND17	136	100	36
PND17/PND21	728	665	63
PND21/PND28	906	838	68

Highest correlation in gene expression differences was detected between sample comparison of PND 17/21 and PND 21/28 (total number of common DE genes 255, Figure 1B), where almost all common DE genes were regulated in the same direction in both comparisons (99%, Figure 1C and D). High number of overlapping (n = 203) and agreeing (98%) DE genes were also present in sample comparisons of PND 7/14 and PND 17/21 indicating a change in the transcription of same genes at early spermatocyte and spermatid level (Figure 1B–D). Comparison of PND 14/17 appeared to have more disagreeing DE genes when compared to other comparisons. Also a low number of shared genes with other comparisons were detected, probably due to the low total number of DE genes in this group. Different DE genes appeared also to be present in comparison of PND 7/14 and 21/28, which is expected. The numbers of overlapping differentially expressed features between the comparisons are presented in figure 1B, agreeing upregulated genes in Figure 1C and downregulated genes in 1D.

At gene level highest ranked genes included several ribosomal and mitochondrial genes and non-coding RNAs, which expression was high in all sample groups. However, the amount of highly expressed testis specific genes and the level of expression increased during the progress of spermatogenesis. The twenty highest expressed genes at each time point are shown in Table S1 and the full gene lists have been submitted to GEO-database (http://www.ncbi.nlm.nih.gov/geo/) with accession number GSE39970.

In our experimental setup, the whole testis RNA was used for sequencing, and the produced data includes both germ cell and somatic testicular cell derived transcripts. In order to understand the contribution of somatic cells in the gene expression differences, we analysed the expression patterns of previously identified SC and Leydig cell age specific genes [22], [23]. Very low expression of SC specific genes *Pla2g4a, Gap43* (Figure S2A) and no expression for *Pap3* were identified and no change in the expression pattern was detected. In addition, expression of SC secreted anti-Müllerian hormone (*Amh*), follicle-stimulating hormone receptor (*Fshr*) and SC specific cancer-associated genes [22] showed low level expression that decreased during the first wave of spermatogenesis, possibly reflecting the increase in germ cell population (Figure S2A). The analysis of gene expression of previously identified Leydig cell specific genes [24], [25] showed some differences in post-natal gene expression pattern (Figure S2B). Two of the analyzed steroidogenic genes (*Star and Vcam1*) had highest expression at PND17 and Cytochrome P450 genes *Cyp11a1* and *Cyp17a1* at PND 28. Development of the adult population of Leydig cells occurs after PND 10 and the expression of steroidogenic genes increases reaching adult levels in the mouse by PND 25 [25], [26]. Thus, it is possible that some of the identified gene expression differences derive from somatic cells.

### Functional Analysis

**Figure 2:**
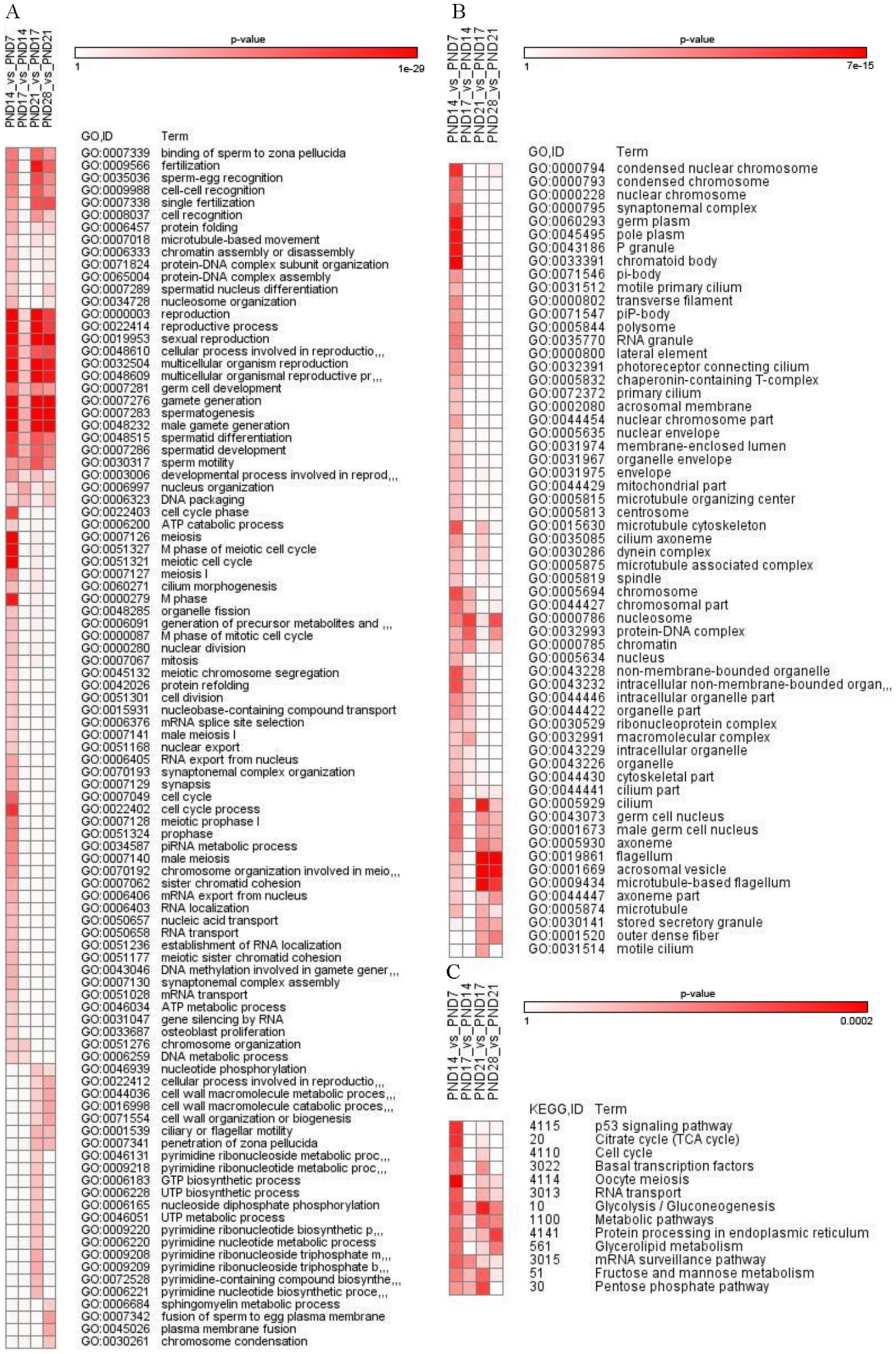
Highest ranked GO terms (p<0.01) with differential expression between sample groups for GO biological processes (A) and cellular components (B) and for KEGG pathways (C). The relative significance of term enrichment is indicated by levels of red shading (the lower the p-value the darker shade of red colour). Heat map was produced with GENE-E (http://www.broadinstitute.org/cancer/software/GENE-E/) using pearson’s correlation metrics and average linkage distance metrics.

TopGO and GOstats R/Bioconductor packages were used for enrichment analysis of the differentially expressed filtered gene lists towards KEGG pathways and Gene Ontology categories. In general, a high number of enriched biological processes and cellular components were found. Analysis of molecular function GO terms resulted with only few significant enrichments. In total 97 GO terms under biological processes were significantly (p<0.01) enriched during the first wave of spermatogenesis (Figure 2A). Although minor part of the differential gene expression may come from the somatic cells (Figure S2), germ cell development associated terms were highlighted in the enrichment analysis. DE genes during the transition from spermatogonia to early spermatocytes (PND7_vs_PND 14) were highly enriched in biological processed involved in mitotic and meiotic pathways. Also expression of genes involved in RNA processing and transport were significantly altered. During meiotic progress (PND 14_vs_PND 17) genes involved in pathways for chromosome organization and nucleus packing were further highlighted. At the onset of post-meiotic spermatid phase (PND 17_vs_PND 21) cell wall associated pathways, pyrimidine processing and ciliary motility were highlighted. Finally, the transition from round to elongating spermatids induced differential expression of genes important for fusion of sperm to egg. Throughout the first wave of spermatogenesis enriched GO terms in biological process were associated with spermatogenesis, reproduction, meiosis and fertilization (Figure 2A).

Enriched GO terms under cellular components included 60 different terms (p<0.01, Figure 2B). Between PND 7 and 14 gene expression in RNA processing associated with chromatoid body, pi-body, p-granule and RNA granule was altered. Also microtubule and cilia related GO terms were enriched already in early spermatocytes (PND14) including centrosome, microtubule organizing center, microtubule cytoskeleton, primary cilium and dynein complex. Furthermore, GO terms related to chromosome organization appeared to be enriched already in early spermatocytes (Figure 2B). Nucleosomal genes were also differentially expressed already in early spermatocytes and further in late spermatocytes and elongating spermatids. Enriched GO terms between PND 17 and 21 included cilium, flagellum and acrosomal vesicle related terms, which were further enriched between PND 21 and 28 consistent with the sperm tail and acrosome formation in spermatids.

Our analysis of the enrichment of DE genes in KEGG pathways revealed 13 pathways, which were enriched already between PND 7 and 14. p53 signalling, TCA cycle, cell cycle, basal transcription factors, oocyte meiosis and RNA transcription were mainly enriched in early spermatocytes (Figure 2C). mRNA surveillance pathway and glycolysis were enriched in all comparisons, but more significantly between PND 7/14 and 14/17 for mRNA and 7/14 and 17/21 for glycolysis. Glycerolipid metabolism and protein processing in endoplasmic reticulum were highly enriched between PND 7/14 and PND 21/28. Fructose and mannose metabolism and pentose phosphate pathway were enriched until PND28. In addition, metabolic pathways were enriched throughout the first wave of spermatogenesis (Figure 2C).

### Functional Comparison of Up- and Downregulated Genes

Enrichments to GO terms were also analyzed separately for up- and downregulated genes in order to identify direction of gene expression in different terms. This analysis was carried out using GOrilla web tool [20], [21]. Figure S3 shows the overall distribution of identified GO terms among up- and downregulated gene lists. Consistently with the high number of upregulated genes in each comparison, most of the enriched GO terms of DE genes were identified in the upregulated gene groups. However, in the comparison of PND 14/17 enriched GO terms were statistically significant (p<0.01) only in the downregulated genes. In this comparison, almost all upregulated genes were non-coding or spliceosomal RNA genes (Table S2).

**Figure 3:**
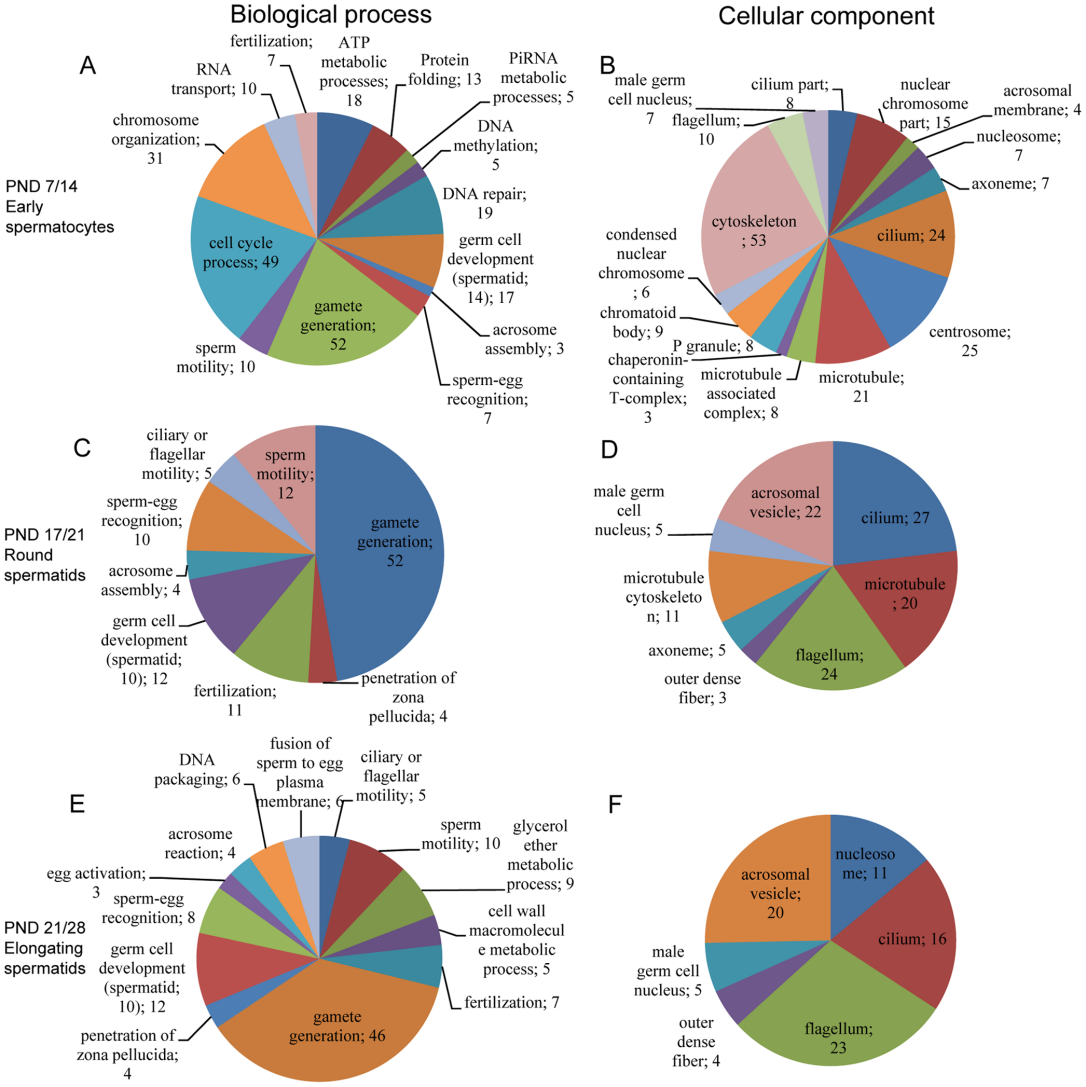
Enriched biological process and cellular component GO terms among upregulated genes. Comparison of PND14/17 is not presented due to low number of DE genes. A. PND 7/14 biological processes, B. PND 7/14 cellular component, C. PND 17/21 biological processes, D. PND 17/21 cellular component, E. PND 21/28 biological processes and F. PND 21/28 cellular component. Only lower level GO terms are shown.

As most of the DE genes were upregulated, also the GO terms identified within the upregulated DE genes corresponded with the GO terms identified within the full set of DE genes (Figure 3). Gamete generation, germ cell development, sperm-egg recognition, fertilization and sperm motility related GO terms were upregulated in all comparisons (except PND 14/17) (Figure 3A,C and E). GO terms specific for spermatogonia early spermatocyte transition were cell cycle processes, chromosome organization, RNA transport, DNA metabolic processes (methylation and repair) and ATP and piRNA metabolic processes. Also, specific GO terms were identified for spermatid elongation phase between PND 21 and 28 and were mainly related to fertilization; egg activation, acrosome reaction and membrane fusion. Enriched cellular component terms of upregulated genes correlated with the BP terms (Figure 3B,D and F). The identified gene expression changes are consistent with the developmental processes in male germ cell maturation. Venn diagrams for sample group comparisons with enriched biological process and cellular component GO terms for upregulated genes are shown in figure S4.

**Figure 4:**
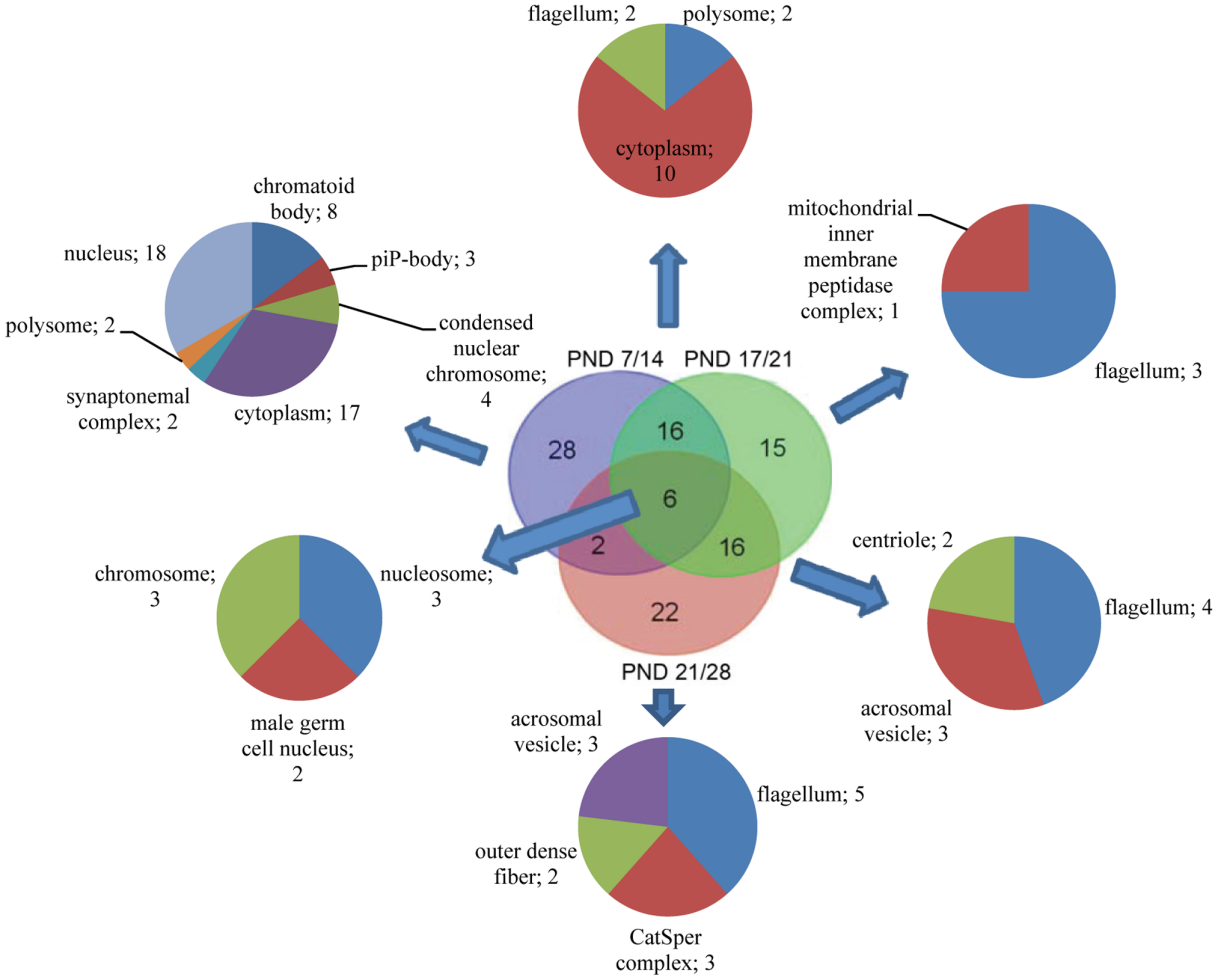
Venn diagram and enriched cellular complex GO terms of upregulated genes in biological process GO term gamete generation. Only lower level GO terms are shown.

GO terms, which were enriched in all sample group comparisons (except PND 14/17) were investigated at gene level in order to see, if same genes are up or downregulated at different time points. Some of the upregulated genes were detected in several sequential group comparisons, but in general, highly specific gene expression was identified between the specific time point comparisons. PND 7/14 and 21/28 appeared to have the most different upregulated gene expression patterns (Figures 1 and 4). We analyzed the DE genes enriched under the GO term gamete generation more carefully and showed that developmental phase specific genes were upregulated in each comparison (Figure 4). The cellular compartment GO terms identified for these genes demonstrated that genes specifically upregulated in gamete generation between samples PND 7 and 14 (n = 28) were localized to nucleus, cytoplasm, chromatoid body, piP body, polysome, synaptonemal complex and condensed nuclear chromosome (Figure 4). Specific upregulated genes between PND 17 and 21 (n = 15) were localized in the flagellum and mitochondrial inner membrane peptidase complex and in comparison of PND 21/28 (n = 22) in the acrosomal vesicle, CatSper complex, flagellum and outer dense fiber (Figure 4). Genes upregulated in all comparisons (n = 6, *Prm1, Tnp2, Tcp11, Spata24, Spata19* and *Tnp1*) were associated with nucleus, nucleosome and chromosome.

### Gene Variant Analysis

**Figure 5:**
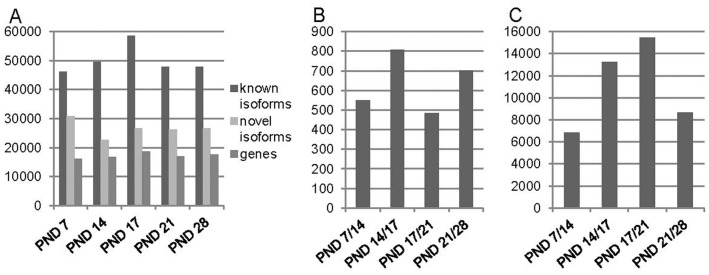
Gene and isoform counts and promoter and transcription start site usage in sample groups. A. Expressed gene, known and novel isoform counts at different time points during the first wave of spermatogenesis. FPKM >1. B. Differential promoter usage in all sample group comparisons. C. Differential transcription start site usage between all sample groups. The comparisons in chronological order during the first wave of spermatogenesis are indicated in black (B and C).

Gene variant detection and differential transcript expression analysis was carried out using the Cufflinks pipeline [19]. On average 17 000 genes, 50 000 known isoforms and 25 000 novel isoforms were expressed in each sample group (FPKM>1, Figure 5A). Differential promoter and transcription start site usage were also analyzed between all sample groups highlighting 500–800 and 7 000 - 15 000 variants between sample comparisons, respectively (Figure 5B and C). Although the differential gene expression analysis with Limma showed a low number of DE genes between PND 14 and 17, a high difference in promoter and transcription start site usage was detected with Cufflinks (Figure 5B and C).

The comparison between transcripts identified by Cufflinks and the Ensembl reference transcripts elucidated the previously unknown splicing events compared to known transcripts. Most of the Cufflinks transcripts (46%) had a complete match with a reference transcript (Table 3). 28% of the identified transcripts were described as potentially novel isoforms and 25% had no match in gene or transcript level to the reference transcripts. Exonic overlap with reference on the opposite strand was found in 937 transcripts (Table 3), which may correspond to potential antisense regulators of sense transcripts. Similar distribution of isoforms was detected for each sample group separately (data not shown). In total 161 362 transcripts were identified in the mouse testis.

**Table 3:** Description of identified gene isoforms classes.

Description	Transcripts	%
Exonic overlap with reference on the opposite strand	937	0.6
Generic exonic overlap with a reference transcript	692	0.4
Potentially novel isoform: at least one splice junction is shared with a reference transcript	45580	28.2
Complete match	74122	45.9
No reference transcript overlap	40030	24.8
Total number of transcripts	161362	100

**Figure 6:**
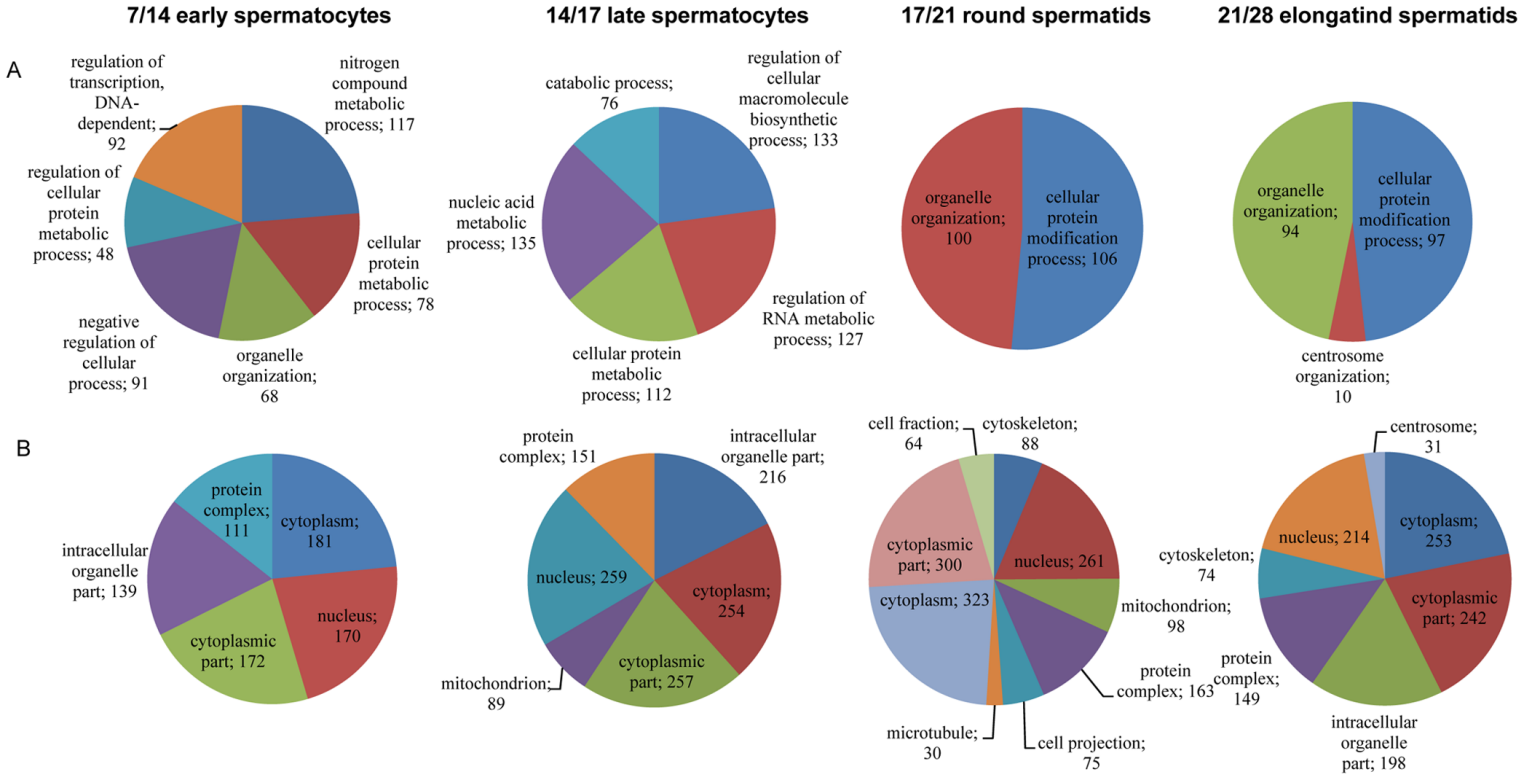
Enriched biological process (A) and cellular component (B) GO terms of genes with differential promoter usage between sample groups (p<10^−6^). Only lower level terms are shown.

The GO term enrichment of the differentially expressed isoforms generated by differential promoter usage were analyzed using GOrilla with differences declared at high significance level (p<10^−6^). Genes with differential promoter usage were associated with molecular function GO terms catalytic activity and binding (protein, ion, nucleotide) in all comparisons. On the whole, general biological processes were enriched within genes related to differential promoter usage (Figure 6A). Genes associated with enriched biological processes at each time point are presented in table S3. Corresponding enrichment pattern was also detected in cellular component GO terms (Figure 6B). Cytoplasm, nucleus, cytoplasmic part and protein complex were enriched in all comparisons. Genes associated with mitochondria were enriched between PND 14/17 and 17/21. Microtubule and cell projection related genes were enriched between PND 17/21 and cytoskeleton genes between PND 17/21 and 21/28. Finally, genes localized to centrosome were enriched between PND 21 and 28 (Figure 6B). Centrosome associated genes were involved in centrosome duplication, spindle organization, mitosis, protein localization, regulation of microtubule cytoskeleton organization and cilium assembly.

In total 81 genes were identified as differentially expressed specifically at isoform level (Table S4). Six of the identified genes (total n = 12) at early spermatogenesis were associated with protein binding (Table S4). Most of the DE isoforms (n = 23, not assigned *Zfp706, Usp9x* and *Ifnar1*) between PND 14 and 17 were involved in cellular processes and seven of these genes were associated with protein domain specific binding, nine with nucleotide binding and two with telomeric DNA binding (Table S4). DE isoforms between PND 17/21 and 21/28 did not show any significant enriched processes or functions. Although the expression pattern of isoforms generally correlated with the gene level expression patterns, a high number of isoforms identified in this analysis and differential expression patterns of some specific isoforms provide evidence for the complexity of gene expression in male germ cells.

### Expression of Non-coding RNAs

For identification of long non-coding RNAs (lncRNAs) the colorspace reads were mapped against the NONCODERv3 lncRNA dataset using CLC Genomics Workbench (v.5.5.1). In total close to 30 000 ncRNAs were identified as expressed in the analysed sample groups. The ncRNAs with highest expression in all sample groups were mainly ribosomal RNAs with some protein transport (SRP_7SL), transcription elongation related (7SK) and mitochondrial RNAs (AK131596) (Table S5).

**Figure 7:**
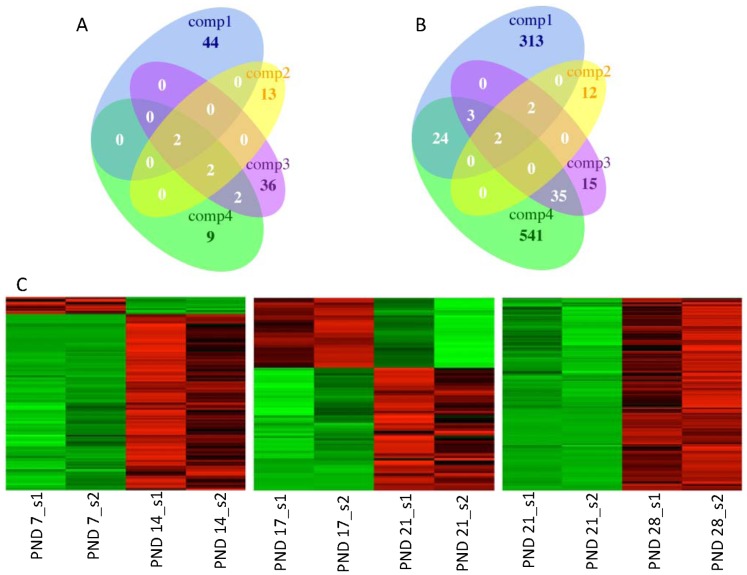
Differentially expressed lncRNAs. A. Venn diagram for all identified downregulated DE lncRNAs. B. Venn diagram for all identified upregulated DE lncRNAs. Comp1 =  PND7/14, Comp2 =  PND14/17, Comp3 =  PND17/21, Comp4 =  PND21/28. C. Heat maps for filtered (p<0.01, FC >2) DE lncRNAs between comparisons. Upregulated genes are indicated in red and downregulated in green.

The differential expression analysis of lncRNA highlighted specific groups of up- and downregulated genes at each time point during the first wave of spermatogenesis. In total of 108 downregulated (Figure 7A) and 947 upregulated lncRNAs (Figure 7B) were identified with high correlation between samples of the same group (Figure 7C). Highest number of upregulated DE lncRNAs was identified at critical phases of spermatogenesis during the appearance of spermatocytes (PND 7/14) and spermatid differentiation (PND 21/28) (Figure 7B). The highest number of down regulated genes was detected during early spermatogenesis (PND 7/14), and also between PND 17 and 21 during the transition from the meiotic to the post-meiotic program (Figure 7A). The downregulation fold change (FC) was low, although some statistically significant (p<0.01, FC >2) DE lncRNAs were identified (Figure 7A). Upregulated lncRNAs showed higher FCs and expression providing an interesting set of novel DE lncRNA genes during spermatogenesis (Figure 7B, Table S5). Most of the identified lncRNAs had no known function. Thus, the elucidation of the roles of these lncRNAs requires additional studies.

## Discussion

The first wave of spermatogenesis in the mouse provides an invaluable tool for characterization of gene expression and cellular events during spermatogenesis. Analyzing cells at their physiological environment eliminates the possible changes in expression level due to preparation procedures. However, the heterogeneity of tissue samples needs to be taken into account when interpreting the results. In this study we have extracted testis samples at five different time points during the first wave of spermatogenesis. The cell content in each sample was confirmed with squash preparations (Figure S1) and corresponded to expected distributions of the most mature cell types in the seminiferous epithelium: spermatogonia at PND 7, early pachytene spermatocytes at PND 14, late pachytene spermatocytes at PND 17, round spermatids at PND 21 and elongating spermatids at PND 28. In addition all samples contained somatic cells such as Sertoli and Leydig cells, which also contribute to the identified expression patterns and cannot be entirely distinguished from the gene expression in germ cells. The analysis of known genes expressed by SCs indicated that some of the DE downregulated genes may originate from these somatic cells. SCs differentiate until PND 15 and their gene expression also changes based on germ cell content in the seminiferous tubules [27]. However, only very low expression levels of SC specific genes were identified in the dataset and no expression difference was seen in previously reported DE Sertoli cell genes [22], [23]. The analysis of Leydig cell specific gene expression showed differential expression for some of the analyzed genes. The steroidogenesis takes place between PND 11 and 35 in the mouse testis inducing the expression of steroidogenic genes in the Leydig cells [25], [26]. However, the differential expression between consecutive groups was only detected for Cytochrome P450 genes *Cyp11a1* and *Cyp17a1.* Thus a minor part of the identified differential gene expression appears to come from somatic cells in addition to germ cells.

The Ensemble database contains 37 315 annotated mouse genes (including protein-coding genes, long noncoding RNA genes, pseudogenes, and short noncoding RNA genes), of which we identified 26 789 genes in the mouse testis. This indicates that 72% of all annotated genes are expressed in the testis. In total 2494 DE genes were identified in the comparisons between the consecutive time points, thus 9% of identified genes were differentially expressed. However, in this study we used stringent filtering cutoffs in order to minimize the amount of false positive results and the DE gene counts especially at lower fold change level are substantially higher with less stringent cutoffs. Functional analysis of DE genes revealed enriched GO terms connected to spermatogenesis related processes such as meiosis, piRNA metabolism, nucleus condensation and flagella formation further supporting the correct experimental design. Genes involved in gamete generation, germ cell development, fertilization and sperm motility were upregulated throughout the first wave of spermatogenesis. However, specific set of genes for each GO term appeared to be upregulated in different time point comparisons. Comparisons of GO terms between different time points highlighted the processes and cellular structure characteristics for each phase of spermatogenesis. Cell cycle processes, chromosome organization, RNA transport, protein folding and piRNA processes were specifically upregulated during the transition from spermatogonia to early meiotic cell (PND 7 to PND 14). Acrosome reaction and sperm/egg fusion related terms were enriched during postmeiotic spermiogenesis (PND 21 to PND 28). Only a low number of genes were downregulated in analyzed comparisons. The identified low fold change downregulation may also be due to increase in total gene transcript amounts during progression of spermatogenesis. However, enriched GO terms between samples PND 14 and 17 were only found for downregulated genes. These included mRNA polyadenylation, chromosome condensation and sperm motility and were related to cellular components nucleosome, nucleus and chromosome.

Previous microarray studies have provided valuable information regarding genes involved in spermatogenesis [7], [10], [16], [28]–[33]. These studies have indicated the specificity of gene expression during spermatogenesis and possibility to find biomarkers for male infertility. Although microarray experiments have provided some interesting results, the comprehensive understanding of gene expression affecting sperm formation and male fertility requires a deep sequencing approach, which allows the identification of previously unknown transcripts as well as isoform detection.

Large scale sequencing has enabled discovery of different splicing events and highlighted the high diversity and incidence of different gene isoforms. Previous RNA-seq studies in the human have suggested, that transcripts from approximately 95% of multiexon genes undergo alternative splicing and that there are approximately 100,000 intermediate to high abundance alternative splicing events in major human tissues [34]. We identified a large number of gene isoforms in the testis during the first wave of spermatogenesis. Compared to the identified gene count (∼15 000, FPKM>1) a considerably high number of gene isoforms (>70 000, FPKM>1) were detected with the Cufflinks package. In total over 160 000 isoforms were identified of which ∼40 000 did not have any reference overlap. Approximately 30% of gene assigned transcripts were characterized as possible novel isoforms and 57% of all expressed genes had at least two isoforms.

The high isoform count indicates that differences in gene isoforms explain part of the specific events during spermatogenesis. Samples PND 14 and 17 did not have a high number of DE genes in the gene level analysis. However, the isoform analysis showed a considerable number of differential promoter and transcription start site usage events between PND 14 and 17. Thus, the meiosis related differential expression of genes may be more influenced by isoform differences and low level of DE at gene level. In addition, the analysis of lncRNA based on Noncode database highlighted phase specific expression of lncRNAs. Since these lncRNAs have a lower level expression, the comprehensive analysis of these genes requires deep sequencing of enriched cell population samples. Based on this study, it is clear that the identification of novel and tissue specific gene isoforms and ncRNAs is crucial for the comprehension of the complexity of transcriptomes and gene regulation. Our data elucidates the diversity of expressed genes and isoforms during spermatogenesis and lays the ground for further studies of specific factors contributing to male fertility.

## Supporting Information

Figure S1 **Validation of the cell content in the testis tissue samples.** A. Squash preparations of the samples at PND14, 17, 21 and 28. Each sample at specific time point contained similar cell population. Distinctive cell types of each time point are labeled. ePSc, early pachytene spermatocyte; lPSc, late pachytene spermatocyte; RS, round spermatid; ES, elongating spermatid. B. Testis weights at PND 7, 14, 17, 21 and 28. Very low variation was identified between samples at each time point (SD error bars).
(TIFF)

Figure S2 **Somatic cell gene expression profiles during the first wave of spermatogenesis.** A. Sertoli cell specific gene expression is low and decreases during the appearance of different germ cell populations. B. Leydig cell specific gene expression shows mainly low level expression with few DE genes at PND 28.
(TIFF)

Figure S3 **Enriched GO terms for up- and downregulated DE genes (GOrilla).** Most of the enriched GO terms were identified with upregulated genes except for comparison of PND 14 and 17.
(TIFF)

Figure S4 **Venn diagrams for biological process (A) and cellular component (B) GO terms for upregulated genes.** Most of the enriched GO terms were specific for the spermatogonia/spermatocyte transition. The terms enriched in all comparisons were related to male germ cell development and gamete generation.
(TIF)

Table S1 **Twenty highest expressed genes at different time points during the first wave of spermatogenesis.**
(DOCX)

Table S2 **Upregulated genes in comparison of PND14 and 17.**
(XLSX)

Table S3 **Genes with differential promoter usage associated with specific GO terms.**
(XLSX)

Table S4 **Genes identified specifically as differential expressed at isoform level.** Genes in enriched GO terms are highlighted.
(DOCX)

Table S5 **Upregulated DE lncRNAs in analysed comparisons.**
(XLSX)
