# Supplementary figures and images for: Transcriptome Profiling of the Murine Testis during the First Wave of Spermatogenesis
Source: PLoS One. 2013 Apr 17;8(4):e61558. doi: 10.1371/journal.pone.0061558 (PMC3629203; doi:10.1371/journal.pone.0061558)

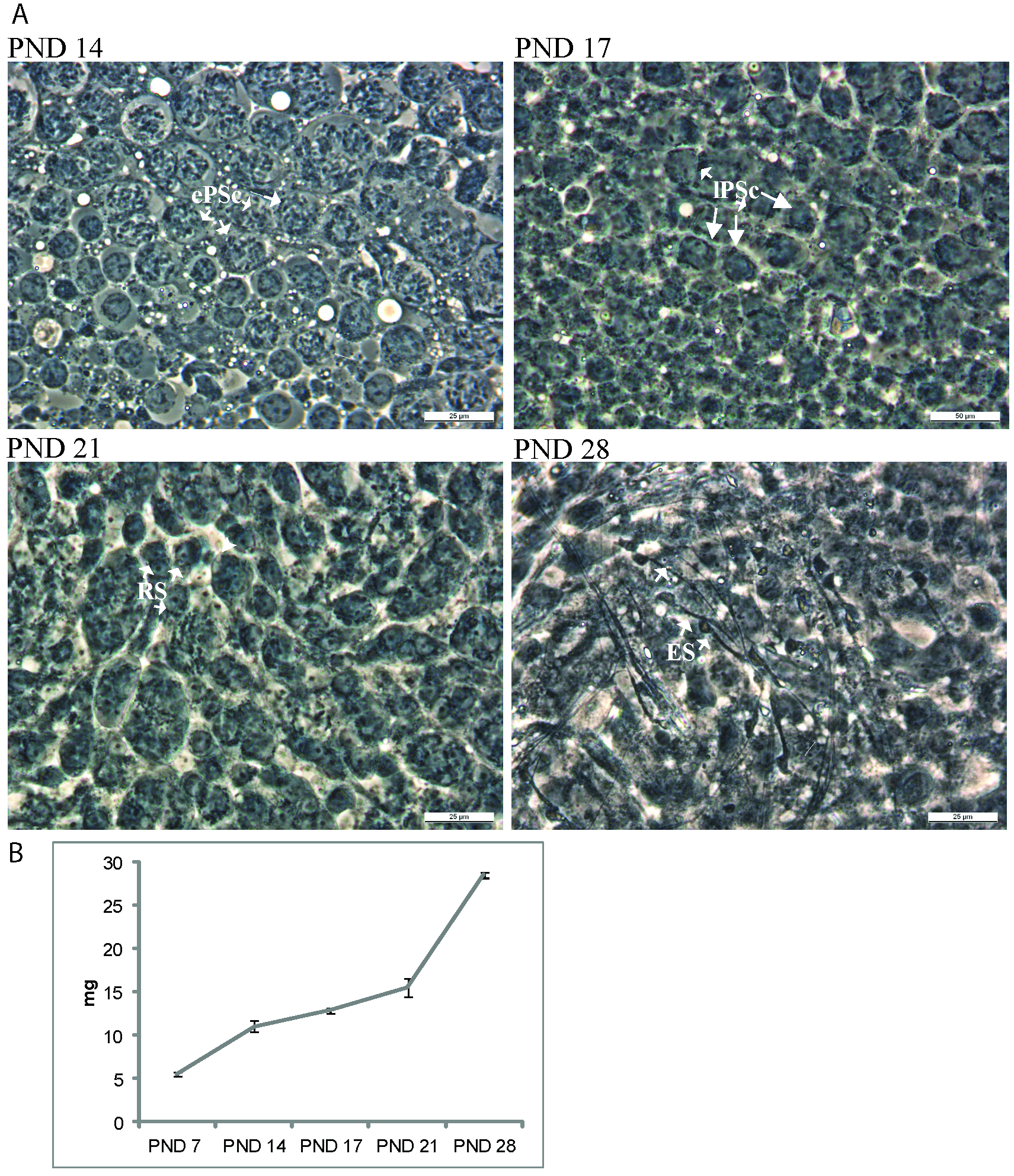

Supplement: Figure S1 — Validation of the cell content in the testis tissue samples. A. Squash preparations of the samples at PND14, 17, 21 and 28. Each sample at specific time point contained similar cell population. Distinctive cell types of each time point are labeled. ePSc, early pachytene spermatocyte; lPSc, late pachytene spermatocyte; RS, round spermatid; ES, elongating spermatid. B. Testis weights at PND 7, 14, 17, 21 and 28. Very low variation was identified between samples at each time point (SD error bars). (TIFF) [file pone.0061558.s001.tiff]

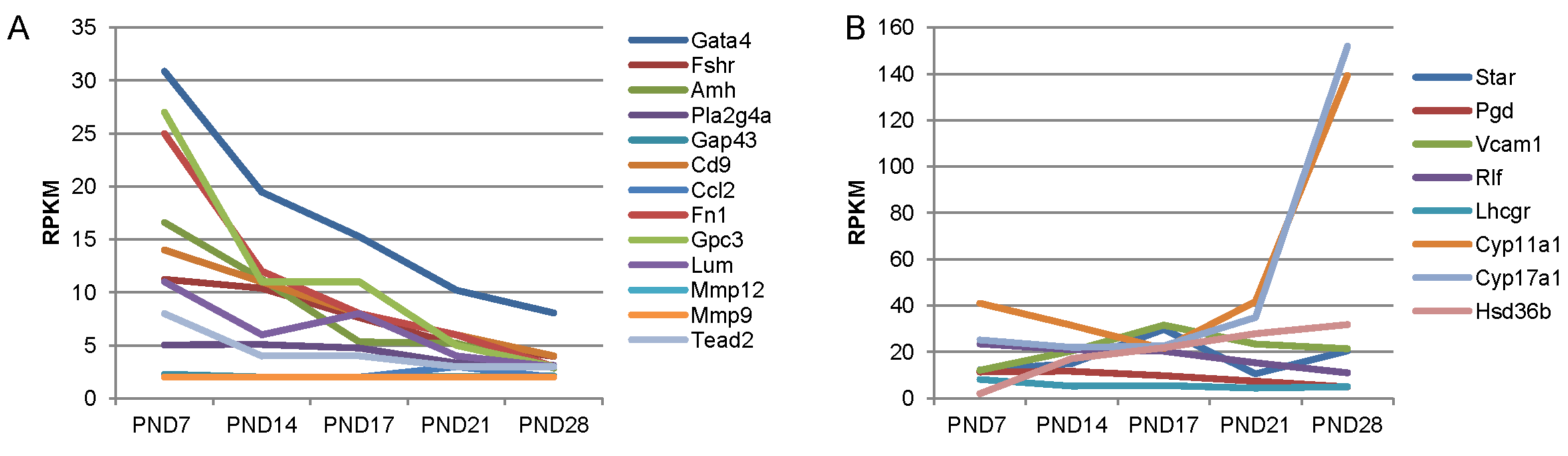

Supplement: Figure S2 — Somatic cell gene expression profiles during the first wave of spermatogenesis. A. Sertoli cell specific gene expression is low and decreases during the appearance of different germ cell populations. B. Leydig cell specific gene expression shows mainly low level expression with few DE genes at PND 28. (TIFF) [file pone.0061558.s002.tiff]

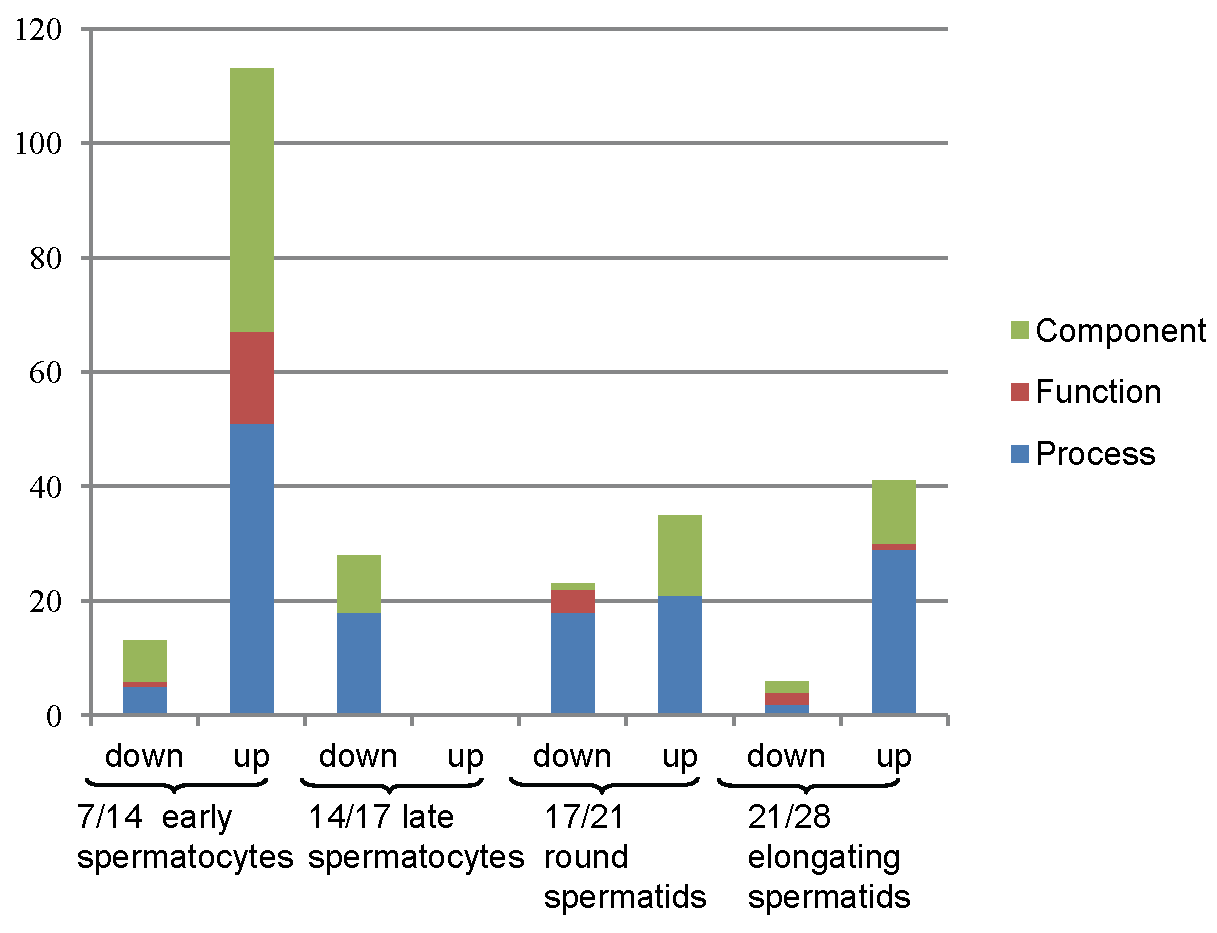

Supplement: Figure S3 — Enriched GO terms for up- and downregulated DE genes (GOrilla). Most of the enriched GO terms were identified with upregulated genes except for comparison of PND 14 and 17. (TIFF) [file pone.0061558.s003.tiff]

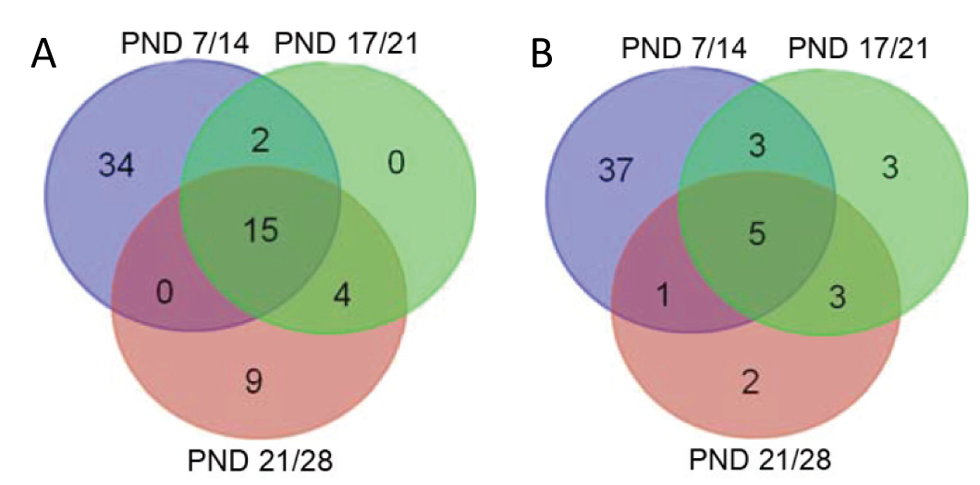

Supplement: Figure S4 — Venn diagrams for biological process (A) and cellular component (B) GO terms for upregulated genes. Most of the enriched GO terms were specific for the spermatogonia/spermatocyte transition. The terms enriched in all comparisons were related to male germ cell development and gamete generation. (TIF) [file pone.0061558.s004.tif]
