# Supplementary material for: Transcriptome Profiling of the Murine Testis during the First Wave of Spermatogenesis
Source: PLoS One. 2013 Apr 17;8(4):e61558. doi: 10.1371/journal.pone.0061558 (PMC3629203; doi:10.1371/journal.pone.0061558)
Supplement: Table S1 — Twenty highest expressed genes at different time points during the first wave of spermatogenesis. (DOCX) [file pone.0061558.s005.docx]

| **PND7** | **PND14** | **PND17** | **PND21** | **PND28** |
| --- | --- | --- | --- | --- |
| mt-Rnr2 | mt-Rnr2 | mt-Rnr2 | mt-Rnr2 | mt-Rnr1 |
| mt-Rnr1 | 7SK | RNase_MRP | mt-Rnr1 | RNase_MRP |
| RNase_MRP | Y_RNA | mt-Rnr1 | RNase_MRP | Tnp1 |
| AC102570.1 | RNase_MRP | mt-Tm | mt-Tm | Ldhc |
| mt-Co1 | mt-Rnr1 | Hist1h2ba | mt-Tl1 | mt-Tm |
| mt-Nd1 | mt-Tm | mt-Tl1 | Hist1h2ba | Tnp2 |
| U4atac | Hist1h2ba | Hist1h1a | Ldhc | Prm1 |
| mt-Cytb | Hist1h1a | Snhg1 | mt-Co1 | Fabp9 |
| Malat1 | mt-Tl1 | mt-Co1 | mt-Nd1 | Hist1h2ba |
| mt-Tl1 | mt-Co1 | Rps8 | mt-Tv | Gstm5 |
| mt-Tm | mt-Nd1 | mt-Nd1 | Fabp9 | Fhl4 |
| Gas5 | Snhg1 | Gas5 | mt-Cytb | Crisp2 |
| Hist1h1a | mt-Cytb | mt-Tv | Mael | Spata4 |
| mt-Nd2 | Gas5 | Ldhc | Fhl4 | Mael |
| Snhg1 | AC087117.3 | Defb19 | Hist1h1a | mt-Tl1 |
| Tmsb4x | SCARNA6 | mt-Cytb | Phf7 | Phf7 |
| mt-Tv | Gm12260 | Cst9 | Gstm5 | Meig1 |
| Hist1h2ba | mt-Tv | Hist1h1t | Spata4 | Dbil5 |
| mt-Nd4 | Rps8 | Hspa5 | Crisp2 | Ropn1l |
| Aard | Defb19 | Rps3 | Rps8 | Csda |
