# Supplementary material for: Transcriptome Profiling of the Murine Testis during the First Wave of Spermatogenesis
Source: PLoS One. 2013 Apr 17;8(4):e61558. doi: 10.1371/journal.pone.0061558 (PMC3629203; doi:10.1371/journal.pone.0061558)
Supplement: Table S4 — Genes identified specifically as differential expressed at isoform level. Genes in enriched GO terms are highlighted. (DOCX) [file pone.0061558.s008.docx]

| **PND7/14** | **PND14/17** | **PND17/21** | **PND21/28** |
| --- | --- | --- | --- |
| Lasp1 | Slc30a1 | Nudt4 | Arpc5 |
| Smc6 | *Hnrnph1* | Entpd4 | Kdelc1 |
| Erbb2ip | *Smg6* | Sqle | Usp34 |
| Ngly1 | Atad5 | Rai14 | Ppp2ca |
| Eif4g1 | Acly | Phyhd1 | Tmed4 |
| Eif4a2 | Fut8 | Piwil1 | Pex13 |
| Brwd1 | Arf4 | Stk33 | Med24 |
| Ythdf1 | Basp1 | Myo9a | Nploc4 |
| Skil | Ywhaz |  | Cenpp |
| D4Wsu53e | Zfp706 |  | Arf4 |
| Ints8 | Ifnar1 |  | Rnf17 |
| Rras2 | Odf2 |  | Entpd4 |
|  | Prpf6 |  | Oxr1 |
|  | Eps15 |  | Rpl35a |
|  | Zfp292 |  | Gtf2e1 |
|  | Vcp |  | H2-K1 |
|  | Alg2 |  | Znrd1 |
|  | Kit |  | Atg12 |
|  | Hnrnpd |  | Actr1a |
|  | Dusp11 |  | Fam107b |
|  | Kras |  | Cdan1,Ttbk2 |
|  | C80913 |  | Smc4 |
|  | Adam32 |  | Exosc10 |
|  | Usp9x |  | Eif3i |
|  | Gata1 |  | Pmpcb |
|  | Acsl4 |  | Cdkl2 |
|  |  |  | Chchd2 |
|  |  |  | Necap1 |
|  |  |  | Tra2a |
|  |  |  | Rnf181 |
|  |  |  | Uba3 |
|  |  |  | Xrra1 |
|  |  |  | Oat |
|  |  |  | 6430531B16Rik |
|  |  |  | Siah1a |

| protein binding |
| --- |
| protein domain specific binding |
| nucleotide binding |
| *telomeric DNA binding* |
